# Capture and discard practises associated with an ornamental fishery affect the metabolic rate and aerobic capacity of three-striped dwarf cichlids *Apistogramma trifasciata*

**DOI:** 10.1093/conphys/coad105

**Published:** 2024-01-27

**Authors:** Oluwaseun Ojelade, Zoe Storm, Cheng Fu, Daphne Cortese, Amelia Munson, Sarah Boulamail, Mar Pineda, Daiani Kochhann, Shaun Killen

**Affiliations:** Department of Aquaculture and Fisheries Management, Federal University of Agriculture, Abeokuta, Ogun, Nigeria; School of Biodiversity, One Health and Veterinary Medicine, College of Biomedical and Life Sciences, University of Glasgow, University Avenue, Glasgow, UK, G12 8QQ; School of Biodiversity, One Health and Veterinary Medicine, College of Biomedical and Life Sciences, University of Glasgow, University Avenue, Glasgow, UK, G12 8QQ; School of Biodiversity, One Health and Veterinary Medicine, College of Biomedical and Life Sciences, University of Glasgow, University Avenue, Glasgow, UK, G12 8QQ; Laboratory of Evolutionary Physiology and Behaviour, Chongqing Key Laboratory of Animal Biology, Chongqing Normal University, Chongqing 400047, China; School of Biodiversity, One Health and Veterinary Medicine, College of Biomedical and Life Sciences, University of Glasgow, University Avenue, Glasgow, UK, G12 8QQ; School of Biodiversity, One Health and Veterinary Medicine, College of Biomedical and Life Sciences, University of Glasgow, University Avenue, Glasgow, UK, G12 8QQ; School of Biodiversity, One Health and Veterinary Medicine, College of Biomedical and Life Sciences, University of Glasgow, University Avenue, Glasgow, UK, G12 8QQ; Laboratory of Ecology, Department of Biological and Environmental Sciences and Technologies, University of the Salento, S.P. Lecce-Monteroni, 73100 Lecce, Italy; School of Biodiversity, One Health and Veterinary Medicine, College of Biomedical and Life Sciences, University of Glasgow, University Avenue, Glasgow, UK, G12 8QQ; Laboratory of Behavioural Ecophysiology, Center of Agrarian and Biological Sciences, Acaraú Valley State University, 850 Avenue da Universidade, Sobral, Ceará, Brazil, 62040370; School of Biodiversity, One Health and Veterinary Medicine, College of Biomedical and Life Sciences, University of Glasgow, University Avenue, Glasgow, UK, G12 8QQ

**Keywords:** Aerobic scope, Amazon rain forest, anthropogenic stress, fisheries, metabolism, ornamental fish

## Abstract

Fishing causes direct removal of individuals from wild populations but can also cause a physiological disturbance in fish that are released or discarded after capture. While sublethal physiological effects of fish capture have been well studied in commercial and recreational fisheries, this issue has been overlooked for the ornamental fish trade, where it is common to capture fish from the wild and discard non-target species. We examined metabolic responses to capture and discard procedures in the three-striped dwarf cichlid *Apistogramma trifasciata*, a popular Amazonian aquarium species that nonetheless may be discarded when not a target species. Individuals (*n* = 34) were tagged and exposed to each of four treatments designed to simulate procedures during the capture and discard process: 1) a non-handling control; 2) netting; 3) netting +30 seconds of air exposure; and 4) netting +60 seconds of air exposure. Metabolic rates were estimated using intermittent-flow respirometry, immediately following each treatment then throughout recovery overnight. Increasing amounts of netting and air exposure caused an acute increase in oxygen uptake and decrease in available aerobic scope. In general, recovery occurred quickly, with rapid decreases in oxygen uptake within the first 30 minutes post-handling. Notably, however, male fish exposed to netting +60 seconds of air exposure showed a delayed response whereby available aerobic scope was constrained <75% of maximum until ~4–6 hours post-stress. Larger fish showed a greater initial increase in oxygen uptake post-stress and slower rates of recovery. The results suggest that in the period following discard, this species may experience a reduced aerobic capacity for additional behavioural/physiological responses including feeding, territory defence and predator avoidance. These results are among the first to examine impacts of discard practises in the ornamental fishery and suggest ecophysiological research can provide valuable insight towards increasing sustainable practises in this global trade.

## Teaser

We examined physiological effects of discard practises in the ornamental fish trade on three-stripe dwarf cichlids *Apistogramma trifasciata*. Fish exposed to increased capture stress experienced acute increased metabolism and decreased aerobic scope. These findings highlight the importance of ecophysiological research in promoting sustainability in the global ornamental fish industry.

## Introduction

A common feature of many fisheries is the live discard of at least a proportion of the captured fish. For commercial fisheries, individuals are commonly discarded if they are non-target species or unable to be retained due to legislation (e.g. they are of a non-legal size or are out of season (Falco *et al.,* 2022)). In the case of recreational fishing, ‘catch-and-release’ angling is often performed as a conservation strategy, with the assumption that fish can generally recover and survive after being returned to their natural environment after capture (Arlinghaus *et al.,* 2007; Cooke and Schramm, 2007). In many instances, however, physiological disturbance, bodily injuries, and behavioural impairment have all been reported in discarded fish (Meka and Margraf, 2007; Campbell *et al.,* 2010), all of which may contribute to making fish more susceptible to natural stressors and predation after release in both recreational and commercial fisheries (Raby *et al.,* 2014). While there is a growing understanding of the impacts of the physiological stress that fish experience during recreational and commercial food fishing, little is known about the sublethal impacts of discard in ornamental fish species, despite the fact that there may be copious opportunities for the discard of non-target species, phenotypes or sex in ornamental fisheries (Militz *et al.,* 2016). An additional unique element of the ornamental trade is that fish that are retained after capture are done so with the purpose of keeping them alive for sale. Physiological disturbance incurred during capture may lead to mortality occurring during subsequent holding or transport, resulting in increased numbers of individuals ultimately needing to be removed from the wild. An increased understanding of the physiological disturbance that fish experience during capture would therefore provide a basis for reducing stress and improving welfare and survival of fish in the ornamental trade.

The ornamental fish trade is a global fisheries sector that involves the transport and sale of ~6 million tonnes of fish annually (King, 2019; Borges *et al.,* 2021). While this is a relatively low volume compared to other fisheries sectors (Food and Agriculture Organisation (FAO), 2016), the ornamental sector is considered to be of disproportionately high economic value, equating to ~1.3 billion individual fish per year, with an estimated value of US$15–20 billion (King, 2019). Some ornamental fish are bred in captivity, but due to difficulties with breeding in many species, a significant proportion are harvested from the wild (Evers *et al.,* 2019). While the issue of discards has been generally overlooked in ornamental fishery, it is likely to be substantial given that the fishing gear that are used are often non-selective traps or nets that will also capture non-target or non-valued species (Militz *et al.,* 2016). As a result, the direct harvest of fish for the ornamental trade, plus the lethal or sublethal effects of discard practises, may have important but unknown effects that are relevant for ongoing discussions surrounding the environmental impacts of fishing for the ornamental trade. On one hand, there is concern that ornamental fisheries may be unsustainable or involve insufficient animal welfare considerations (Cohen *et al.,* 2013). On the other hand, when done sustainably, ornamental fisheries provide an economic foundation for small communities in developing regions and provide financial incentives for protecting aquatic habitats and their surroundings (Prang, 2001). While this potential conflict should be resolvable, with fish being sustainably harvested in a manner that prioritizes welfare and is profitable for fishers and their communities, there is an absence of information on issues that are fundamental for making informed policies or understanding the ecological impacts of ornamental fishing practises. This includes a basic understanding of how discarded species or individuals may be affected by stressors encountered during capture and their subsequent release.

The procedures involved in the capture of ornamental fish may cause various degrees of physiological or metabolic disturbance in the captured fish. For small ornamental fishes, particularly freshwater species, capture is often performed using traps or handheld nets. While these methods avoid many of the physical injuries that can be caused during large-scale commercial fisheries (e.g. decompression, crushing or other trauma in seines and trawls) or recreational fisheries (e.g. hooking injuries), the handling that fish encounter may still generate important sublethal physiological effects. Burst-type swimming activity or struggling during netting or confinement can increase energy expenditure, and although vigorous movements generally involve anaerobic metabolism, this can cause a subsequent increase in aerobic metabolic rate during recovery (Lee *et al.,* 2003) due to the costs of correcting disturbances to osmoregulatory status, muscle glycogen and lactate concentrations and other physiological changes occurring during exercise (Kieffer, 2000). Notably, these adverse physiological effects of capture-related stressors appear to be most pronounced for larger individuals within species, at least for species targeted in recreational fisheries (e.g. coho salmon, Clark *et al.,* 2012; largemouth bass, Gingerich and Suski, 2012). During the capture of ornamental fish, individuals are generally exposed to air for at least a brief period during initial sorting. The utilization of anaerobic metabolism and associated physiological disturbance can be exacerbated by air exposure in fish, which causes the collapse of gill lamellae and systemic hypoxia due to the prevention of gas exchange with the surrounding environment (Cook *et al.,* 2015). Taken together, the combined effects of netting, air exposure and general handling may also elicit an autonomic stress response that increases energy use and systemic oxygen demand (Schreck and Tort, 2016). While this phenomenon has been examined in commercial and recreational fisheries, where it has been found that recovery of oxygen uptake may take hours after capture-related stressors (Clark *et al.,* 2012; Raby *et al.,* 2015), it has not been considered in ornamental fisheries, in which fish experience potentially experience different degrees of stress and are generally smaller bodied compared to these other fishery sectors.

Aside from a direct increase in energy expenditure, an increase in metabolic rate may also constrain an animal’s aerobic scope for additional physiological functions (Halsey *et al.,* 2018; Fu *et al.,* 2022). Aerobic scope is functionally defined as the difference between an animal’s aerobic metabolic floor (their standard metabolic rate, SMR, the minimal energy required to sustain life in a resting, post-absorptive animal; Chabot *et al.,* 2016) and their metabolic ceiling (maximum metabolic rate, MMR, the maximum rate of aerobic metabolism achievable by the animal; Norin and Clark, 2016), and represents the capacity of the cardiovascular system to deliver oxygen to tissues for aerobic metabolism. Any oxygen use and delivery required by one aerobic function (e.g. physical activity) will, in general, cause a reduction in the capacity to perform others (e.g. digestion; (Clark *et al.,* 2013; Halsey *et al.,* 2018; McLean *et al.,* 2018). As such, a rise in metabolic rate produced during the stress responses to capture-associated stressors in fish may constrain an individual’s available aerobic scope and compromise the ability to perform additional aerobic tasks. While there is evidence that stressors encountered during the catch-and-release or discard of fish can cause an increase in oxygen uptake and a reduction in the available aerobic scope of fishes in recreational and commercial fisheries (Clark *et al.,* 2012; Cooke *et al.,* 2014; Raby *et al.,* 2015), this phenomenon has not been investigated within the context of ornamental fisheries.

**Figure 1:**
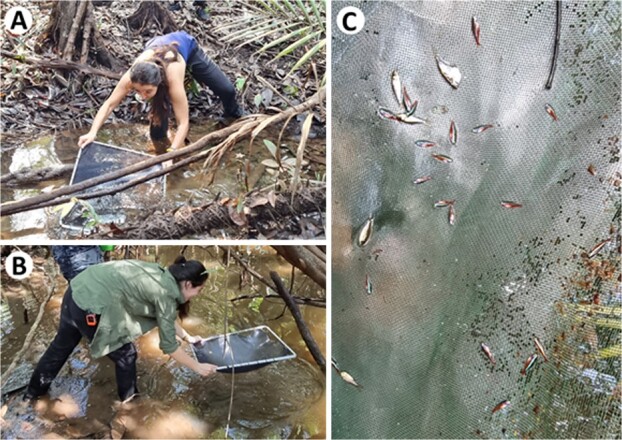
Capture process in an ornamental fishery in the Amazon. (A) A specialized hand net called a *rapiche* is used to capture fish. The net is rapidly plunged through the water to catch fish on and within leaf-litter substrate; (B) Fish contact the net during the capture process; and (C) may experience brief but direct air exposure during sorting. Note that the fish visible in panel C are hatchet fish (exact genus and species unknown) and cardinal tetra *Paracheirodon axelrodi*. Photo credit: Shaun Killen.

Of the >90 species of dwarf cichlids of the genus *Apistogramma*, many are harvested from the wild or cultured for the ornamental fish trade in some capacity (Tribuzy-Neto *et al.,* 2020). They are native to South America and are most diverse in the Amazon floodplains, and the diverse array of species with overlapping ranges and habitats can result in the simultaneous capture of multiple species and non-target species of *Apistogramma* being discarded when not specifically sought by fishers due to market demands and space limitations during capture. Additionally, males are often targeted in ornamental fisheries as sexual dimorphism within the species can be observed, with males being more colourful than females and thus in higher demand in the aquarium trade (Römer, 2021). *Apistogramma* spp. are often captured using a specialized dipnet called a *rapiche* (Fig. 1), after which they may be air-exposed during sorting or transfer to holding containers (~30–60 seconds; Pineda, Kochhann, Killen personal observations). Notably, *Apistogramma* spp. show complex and sexually dimorphic behaviours associated with territoriality, reproduction and parental care. From the perspective of physiological responses to discard, this could be relevant, given that sex can contribute to intraspecific variability in metabolic rates and sensitivity to stressors (Köhler *et al.,* 2011), including during catch-and-release practises in recreational fisheries (Papatheodoulou *et al.,* 2022).

We examined these issues in the three-striped dwarf cichlid *Apistogramma trifasciata*. We hypothesized that if netting and air exposure cause a physiological stress response in captured fish, there would be an increase in metabolic rate relative to a control treatment, and that as the combined stressors (netting plus air exposure) become more intense, the magnitude of the increase in metabolic rate would increase. Correspondingly, any increase in metabolic rates due to netting and air exposure would cause an accompanying decrease in available aerobic scope. To test this hypothesis, we exposed three-striped cichlids to various combinations of netting and air exposure stressors that they encounter during a capture-and-discard event, then measured the immediate change in metabolic rate and throughout recovery using intermittent-flow respirometry.

## Materials and Methods

### Animals

Three-striped dwarf cichlids *A. trifasciata* were purchased from a licenced fish supplier and transported to the University of Glasgow. In the laboratory, fish were divided into two equally sized groups, each of them housed in a glass tank (59 × 37 × 26 cm) filled with recirculating and UV-treated filtered water to 90% of its capacity (30% daily water change). Each holding tank was continuously aerated and enriched with four stands of ornamental plants, wood, aquarium stones (2-cm high), substrate and two PVC tubes (9.5 × 5.0 cm) under a 14-hour light:10-hour dark photoperiod. Fish were acclimatized for 2 weeks before the start of the experiment. The mean water temperature, pH and dissolved oxygen of the holding tanks were maintained at 28.03 ± 0.9°C, 7.3 and 7.8 ± 0.4 mg/l, respectively. Fish were fed tropical flakes for aquarium fish with 46.0% crude protein twice daily during the period of acclimatization. All animal experimental procedures were executed in accordance with the UK’s Home Office guidelines (Project Licence no. 60/4461).

### Tagging and identification

Fish (*n* = 34) were tagged for individual identification using visible implant elastomer (VIE, Northwest Marine Technology Inc.). A combination of four colours (orange, green, red and yellow) were used on four locations along the dorsal area (two on either side). Fish were anaesthetized in 2.5 ml benzocaine per 0.5 l of water before tagging (Zahl *et al.,* 2012). After tagging, all fish were weighed (E14130, OHAUS, Switzerland) to the nearest hundredth of a gram (all fish: 0.564 ± 0.040 g, mean ± SEM; females: 0.666 ± 0.066 g; males: 0.449 ± 0.020 g), and their total length was measured to the nearest hundredth of a centimetre with a precision digital caliper (34.98 ± 0.91 cm; females: 35.83 ± 1.29 cm; males: 33.91 ± 1.26 cm). Fish sex was also determined (18 females, 16 males) at this stage. The entire procedure took less than a minute per fish. A few minutes after the handling procedure, fish resumed normal behaviour and were transferred back to their holding tank where they were monitored for 24 hours.

### Experimental treatments

Seven days post-tagging, at 09:00 in the morning, a subset of cichlids were carefully guided into individual cylindrical perforated plastic containers (12.5 × 7.5 cm; one fish per container) underwater, within their holding tanks, then allowed to rest in the covered containers in their holding tanks for 5 hours (with the containers floating in their holding tanks) before the start of the experiment. Once in the individual plastic containers, the fish could be easily removed from the tanks without netting or air exposure, thus minimizing the effects of capture stress from the holding tanks on the experimental treatments.

All fish were individually exposed in a random order to four treatments simulating four different types of handling stress that they are likely to experience when captured in the wild:

(1) Control. In this treatment, fish were transferred directly from their acclimation container to the respirometer, without air exposure, by gently pouring the contents of the container into the respirometry chamber.

(2) Netting. In this treatment, fish were gently poured from their container into a small bucket, then captured in a small nylon hand net. The fish then remained in the net for 10 seconds before being transferred to the respirometry chamber underwater and without air exposure. There was no specific attempt to chase the fish before netting, as the capture of *Apistomgramma* spp. generally involves rapidly moving the *rapiche* through the water and substrate, capturing fish resting in leaves before they have an opportunity to be chased.

(3) Netting +30 seconds of air exposure. In this treatment, fish were captured with a hand net as in the netting treatment, but then received 30 seconds of air exposure before being placed in the respirometer.

(4) Netting +60 seconds of air exposure. In this treatment, fish were captured with a hand net as in the netting treatment, but then received 60 seconds of air exposure before being placed in the respirometer.

### Estimation of metabolic rates

Once placed in the respirometer, oxygen uptake measurements commenced for each individual using intermittent-flow respirometry (Svendsen *et al.,* 2016). Full respirometry details are listed in Supplementary Table S1. Fish oxygen uptake was used as a proxy of fish whole-body aerobic metabolism (Killen *et al.,* 2021). Respirometry trials started between 14:00 and 16:00 each day then continued overnight. Fish were exposed to one treatment per day, with each fish being allowed to rest in its holding tank for a minimum of 48 hours between treatments. Each day, 11–15 fish were measured using respirometry, with 3–4 being randomly exposed to each of the different treatments on each experimental day, until all fish had been measured in each treatment. Individuals were allowed at least 2 days between repeated measurements. The feeding of the fish was suspended 24 hours prior to the start of each experimental trial to measure metabolic rate during a non-digestive phase.

The respirometer system included oxygen sensors (PyroScience GmbH, Aachen, Germany) and 16 parallel cylindrical glass chambers submerged into a large water basin (79.5 × 59.0 × 21 cm, 56 l of water), maintained at 28.0 ± 0.1°C using a thermostat and sump. Due to slight variation in fish size, a total of 12 small (30.0 ml) and four large cylindrical chambers (58.3 ml) were used. A single fish was placed in each chamber with one of the chambers left empty as a control for bacterial consumption during the experimental procedure. In addition, blank measures of each chamber were conducted without fish before and after each trial run. The respirometry system was supplied with filtered and UV-treated water. All 16 respirometry chambers were intermittently flushed with clean oxygenated water by a set of four pumps, which were automated to flush for 2 minutes followed by a 4-minute closed phase during which dissolved oxygen was automatically recorded every 2 seconds. Water mixing within the respirometer was achieved with the use of a peristaltic pump.

Each cichlid was placed in the respirometry chamber immediately after exposure to one of the handling stress treatments. Visible interaction between the fish was prevented with black corrugated plastic placed in between the chambers. Recording continued overnight and lasted for an average of 21 ± 2 hours (mean ± SD; Supplementary Table S1). The oxygen depletion of empty chambers was carried out for 30 minutes (5 slopes) before putting the fish in the chambers and for 30 minutes after removing them to account for the background oxygen consumption in each chamber (i.e. microbial respiration). The slopes of the decrease in the oxygen values during closed phases were estimated using the FishResp R package (Morozov *et al.,* 2020). The rate of oxygen uptake by the fish was calculated for each individual by multiplying the obtained slopes by the volume of the respirometry chamber after removing the volume of the fish and adjusting for background microbial respiration (Killen *et al.,* 2021).

For each fish, 24 hours after the last treatment for all fish in the study, MMR was estimated by measuring oxygen consumption rate immediately after exhaustion caused by manual chasing (Table 1; (Norin and Clark, 2016). Each fish was chased to exhaustion in a circular tank (32-cm diameter, 3-l volume) for an average of 44.17 ± 1.42 seconds. Chasing ended when the fish would no longer respond to gentle touching by attempting to swim away. Immediately after the chasing protocol, the fish was transferred to the respirometer to measure oxygen consumption. Visual separation between individuals and respirometry procedures and general procedures was carried out as before, but fish were not left in the respirometers overnight.

**Table 1:** Definitions of each metabolic trait estimated for three-striped dwarf cichlids in this study

**Measure**	**Abbreviation**	**Description**
Standard metabolic rate	SMR	Representative of the minimal energy required to sustain life, excluding growth, digestion and activity costs. Taken as the 20th percentile of oxygen uptake measurements within the control trial, excluding the first 5 hours post-stressor and then only including data up to 15 hours after transfer to the respirometers, to avoid confounding effects of morning activity.
Maximum metabolic rate	MMR	An estimate of the maximum achievable rate of oxygen uptake, in this study performed immediately following manually being chased to exhaustion, during recovery. Calculated as the maximum rate of oxygen uptake measured during a rolling 2-minute interval during the first closed respirometer phase following chasing (Prinzing *et al.,* 2021).
Initial metabolic rate	IMR	The maximum rate of oxygen uptake measured following exposure to the experimental treatments. Estimated immediately following exposure to the treatment. Calculated as the maximum rate of oxygen uptake measured during a rolling 2-minute interval during the first closed respirometer phase following treatment exposure (Prinzing *et al.,* 2021).
Aerobic scope	AS	The amount by which an individual can increase its oxygen delivery to tissues to support aerobic metabolism, above that required for maintenance. Estimated as subtracting SMR from either MMR or the highest IMR measured for a given fish, depending on which was the greatest value.
Remaining aerobic scope	AS_remain_	The available aerobic scope remaining for individuals following exposure to a given experimental treatment, due to their rise in oxygen uptake displayed during that time, expressed as a percentage of total aerobic scope. Calculated immediately following each treatment as ((AS − (IMR − SMR)) / AS) x 100. This value was also calculated for each data point throughout the entire MO_2_ measurement period as ((AS − (MO_2_ − SMR)) / AS) x 100, where MO_2_ = oxygen uptake (mg h^−1^) at a specific time post-treatment.
Excess post-treatment oxygen uptake	none	An estimate of the integrated energy use over time above that required for maintenance (in mg O_2_), taken as the area under the curve of MO_2_ versus time, after subtracting SMR from each data point.

### Statistical analysis

Data from the control treatment for each fish was used to estimate their individual SMR, taken as the lowest 20th percentile of oxygen uptake throughout the measurement, excluding the first 5 hours post-stressor and then only including data up to 15 hours after transfer to the respirometers, to avoid confounding effects of morning activity (Table 1). For MMR and initial metabolic rate (IMR), values were taken as the maximum rate of oxygen uptake during a rolling 2-minute regression window during the first respirometer closed phase after chasing or the experimental treatment, respectively (Prinzing *et al.,* 2021). The aerobic scope (AS; Table 1) in mg O_2_ h^−^^1^ of each fish was calculated as the difference between the maximum level of metabolism estimated across all trials (i.e. maximum value obtained for either MMR or IMR) and SMR. The remaining aerobic scope (AS_remain_), after accounting for any treatment-associated change in MO_2_, was also calculated at each time point, and is expressed as a percentage of maximum AS (Table 1). To estimate the overall excess post-treatment oxygen uptake (in mg O_2_), above that required for maintenance in each treatment, the integrated area under the curve of MO_2_ versus time was calculated for each individual using the trapezoid rule (Fornberg, 2021), after subtracting SMR from the MO_2_ measured at each time point.

All statistical analyses were carried out using R version 4.2.1 (The R Foundation for Statistical Computing, 2022). Linear mixed-effect models (LMEs) were used to test the effects of the treatments on energy expenditure and aerobic capacity of the cichlids, using the packages lme4 (Bates *et al.,* 2015) and lmerTest (Kuznetsova *et al.,* 2017). The response variables were IMR, AS_remain_ and excess post-treatment oxygen uptake, while the explanatory variables were treatment, sex and body mass. Additional models were run using all measures of MO_2_ from over a 15-hour period following each treatment, with log_10_(MO_2_) or log_10_(AS_remain_ + 0.1) as the response variables, and log_10_(mass), log_10_(time + 0.1), sex and treatment as response variables. Model assumptions of linearity and homoscedasticity and normality of residuals were verified using visual inspection of residual-fit plots, and some variables (MO_2_, AS_remain_ (with +0.1 added to account for zero values), mass and time (+ 0.1)) were log-transformed in the latter models to conform to model assumptions. Fish ID was included as a random effect in all models to account for repeated measures of individuals across treatments. Initial models included all main effects and interactions, with model selection proceeding as described by Zuur *et al*. (2009). Non-significant interactions were dropped one at a time, starting with those with the lowest *t*-value, but they were left in the model if their removal resulted in a significantly larger Akaike information criterion (AIC) value as estimated by fitting using likelihood ratio tests. Final model parameters were estimated by fitting using restricted maximum likelihood (Supplementary Table S2, S3). The level of significance was set at alpha, α = 0.05. Values in text are presented as means ± SEM.

## Results

**Figure 2:**
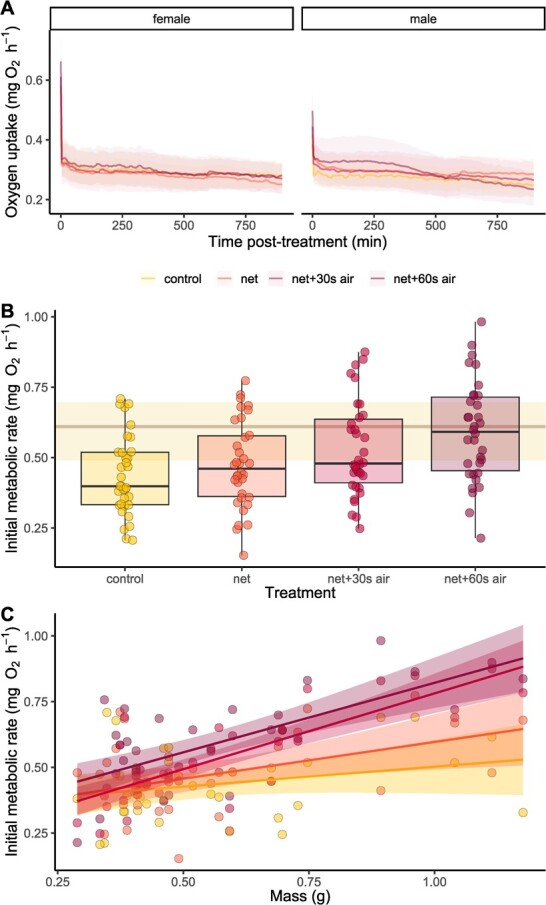
Effects of capture and handling stress: (A) oxygen uptake in the 15 hours following each treatment; (B) the initial metabolic rate immediately following each treatment; and (C) the relationship between initial metabolic rate and body mass in three-striped dwarf cichlids *A. trifasciata* (*n* = 34). In A, the lines connect mean values within each treatment at each 6-minute interval (corresponding to the open–closed phases of the intermittent-flow respirometers); in C, lines represent linear regressions. Shaded areas in A and C represent 95% confidence intervals. Overlaid on the boxplot in B are data points representing one individual. Each individual was tested in each treatment. For panel B, the median MMR for all fish in the study, as elicited by the chase method, is shown for reference and is represented by the horizontal beige line (the shaded area around this line in the range between the 25th and 75th percentiles for MMR). Boxplot lower and upper hinges represent the 25th and 75th percentiles, respectively; the horizontal line within the box represents the median; the length of whiskers represents the range of data points between either the upper or lower hinge and 1.5× the difference between the 25th and 75th percentiles.

Following experimental treatments, most fish showed an immediate increase in oxygen uptake (Fig. 2A), with an increase in IMR in treatments with greater degrees of handling and air exposure (Fig. 2B; LME, effect of treatment, F = 11.19, *P* < 0.0001). Overall, for example, IMR was 35% higher for the netting +60 seconds of air exposure treatment (0.591 ± 0.03 mg O_2_ h^−1^) as compared to the control treatment (0.436 ± 0.024 mg O_2_ h^−1^). Fish with a greater body mass showed a greater IMR and showed the greatest relative increase in IMR in the treatments with air exposure (Figure2C, LME, effect of mass × treatment interaction, 6.15, *P* = 0.0007).

Most of the recovery from the treatments, in terms of a decrease in oxygen uptake over time, occurred within the first 4–30 minutes post-treatment (Fig. 2A). Recovery of MO_2_ was slowest when fish were exposed to netting +60 seconds air exposure, remaining elevated compared to other treatments for ~6 hours post-treatment, then gradually decreasing beyond this time (Fig. 2A; LME, time × treatment interaction, F = 83.62, *P* < 0.0001). Larger individuals showed a slower decrease in MO_2_ over time post-treatment (LME, mass × time interaction, F = 76.76, *P* < 0.0001). Despite this, however, females—which on average were larger than males in our study—recovered MO_2_ more quickly than males, especially in the netting +60 seconds air exposure treatment (Fig. 2A; LME, sex × time × treatment interaction, F = 82.13, *P* < 0.0001). There were no effects of sex or mass on excess post-treatment O_2_ uptake over the entire course of the oxygen uptake measurements (LME, *P* > 0.5 in all cases) or during the first 6 hours post-treatment (when the MO_2_ of the netting +60 seconds air exposure treatment was elevated compared to other treatments; LME, *P* > 0.3 in all cases; Table 2). In the most extreme handling treatment (netting +60 seconds of air exposure), however, excess post-treatment oxygen uptake was 0.367 mg O_2_ after 6 hours, which is 24.14% above that required for maintenance over this time (Table 2), although this effect was not statistically different from the control treatment (LME, effect of treatment, t = 1.746, *P* = 0.083).

**Table 2:** Integrated excess post-treatment oxygen uptake over 6 or 15 hours post-exposure to various treatments simulating capture handling stressors. Excess oxygen uptake was taken as the area under the curve of MO_2_ versus time post-treatment, after subtracting the costs of maintenance (SMR). Also shown is the relative (%) increase in oxygen uptake, relative to what would be required for maintenance over the same time frame.

	**Over 6 hours**		**Over 15 hours**	
	**Excess MO**_**2**_ **(mg O**_**2**_ **)**	**% Above maintenance**	**Excess MO**_**2**_ **(mg O**_**2**_ **)**	**% Above maintenance**
Control	0.225 ± 0.058	15.937 ± 3.892	0.402 ± 0.068	11.721 ± 1.952
Netting	0.230 ± 0.053	17.229 ± 3.747	0.402 ± 0.063	11.290 ± 1.766
Netting + 30 s air	0.211 ± 0.043	15.053 ± 2.526	0.367 ± 0.051	10.722 ± 1.317
Netting + 60 s air	0.367 ± 0.070	24.141 ± 3.892	0.526 ± 0.088	13.636 ± 1.820

**Figure 3:**
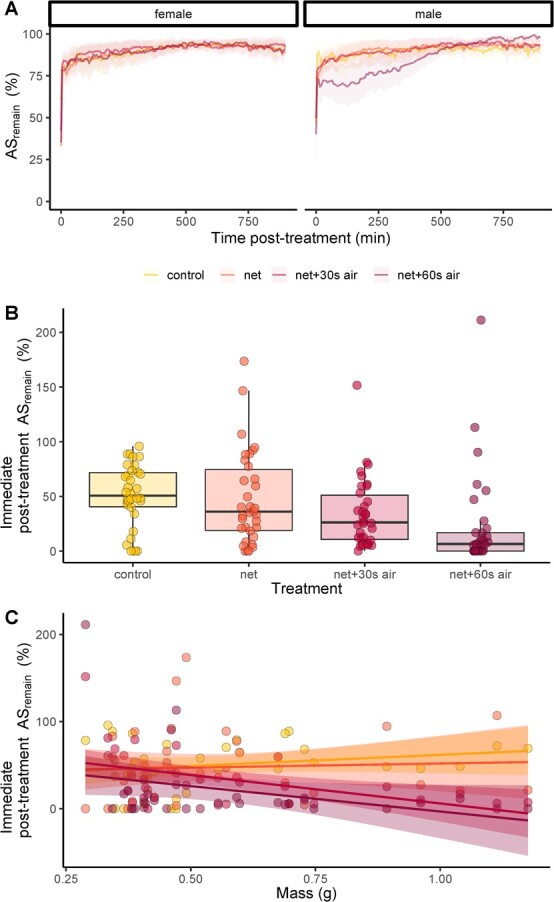
Effects of capture and handling stress on: (A) remaining aerobic scope (AS_remain_) in the 15 hours following each treatment; and (B) AS_remain_ immediately following each treatment; and (C) the relationship between immediate post-treatment AS_remain_ and body mass, in three-striped dwarf cichlids *A. trifasciata* (*n* = 34). In A, the lines connect mean values within each treatment at each 6-minute interval (corresponding to the open–closed phases of the intermittent-flow respirometers); in C, lines represent linear regressions. Shaded areas in A and C represent 95% confidence intervals. Overlaid on the boxplot in B are datapoints representing one individual. Each individual was tested in each treatment. Boxplot lower and upper hinges represent the 25th and 75th percentiles, respectively; the horizontal line within the box represents the median; the length of whiskers represents the range of data points between either the upper or lower hinge and 1.5× the difference between the 25th and 75th percentiles. Data points beyond the extent of the whiskers are outliers.

The increased oxygen uptake after the treatments resulted in a decrease in AS_remain_ (Fig. 3A). Fish experienced a greater reduction in AS with increasingly severe degrees of handling and air exposure (Fig. 3B; LME, main effect of treatment, F = 8.34, *P* < 0.0001). For example, the AS_remain_ of fish immediately following the netting +60 seconds air exposure treatment was on average only 44% of the AS_remain_ for fish immediately after the control treatment, with almost all fish in the netting +60 seconds air exposure treatment approaching the limits of their AS at this time (Fig. 4B). Fish with a greater body mass showed the greatest relative decrease in AS in treatments with air exposure (Fig. 3C, LME, effect of mass × treatment interaction, 3.70, *P* = 0.013). As with MO_2_, the majority of restoration in AS_remain_ occurred within 4–30 minutes post-treatment (Fig. 3A), with fish in most treatments increasing to >75% AS_remain_ during this time. An exception was male fish in the netting +60 seconds air exposure treatment, for which mean AS_remain_ was lower relative to the other treatments and <75%, until ~4 hours post-treatment (Fig. 3A).

## Discussion

We hypothesized that if handling during capture causes a physiological stress response in three-striped dwarf cichlids, fish experiencing netting and air exposure would display an elevation in metabolic rate post-stress relative to a control treatment, with the magnitude of the metabolic response increasing with the severity of the combined stressors. Furthermore, we anticipated that any increase in oxygen uptake due to capture-related stressors would cause a decrease in available aerobic scope. These predictions were generally supported, and although most recovery of MO_2_ and AS_remain_ occurred within 30 minutes post-treatment, there were notable effects of handling stress on metabolic traits that are of potential ecological importance. For example, the aerobic scope of individuals was constrained following exposure to the treatments, with some individuals having little or no aerobic scope for additional oxygen-consuming functions in the minutes following the capture process. These effects were greatest for larger fish when they received increasingly severe stressors (i.e. netting + air exposure). In addition, males recovered more slowly than females, especially when exposed to the most severe handling treatment, with lingering effects of handling stress on MO_2_ and AS_remain_ for several hours post-treatment when receiving 60 s of air-exposure following netting. Therefore, while the acute stressors encountered during capture has a generally modest effect on energy expenditure, there can be a temporary reduction in the capacity for aerobic metabolism that could limit digestion or locomotor activity involved in predator avoidance, foraging or territory defence.

Immediately following simulated capture and discard, individuals showed the greatest increase in oxygen uptake when exposed to a combination of netting stress and air exposure, and this disturbance increased with the duration of air exposure. The proximate cause of the increase in metabolism following handling stress is likely due a combination of: 1) aerobic recovery from any anaerobic metabolism occurring during netting (e.g. due to bursts of anaerobic swimming activity during confinement) or while exposed to air (i.e. due to a decrease in systemic oxygen availability) and 2) activation of an autonomic stress response that can increase oxygen demand. Air exposure has been identified as a primary source of anaerobic metabolism in fish in recreational catch-and-release fisheries (Cook *et al.,* 2015), as the delicate gill lamellae of fish collapse in air and are incapable of gas exchange with the external environment. Aside from a transient increase in metabolic energy expenditure, the most serious consequence of this increased peak level of aerobic metabolism is to constrain the aerobic scope available for other oxygen-consuming physiological processes. In fishes, for example, the metabolic costs of meal digestion and assimilation can occupy a large proportion of an individual’s aerobic scope (Sandblom *et al.,* 2014), so the results of this study suggest that digestion may be impaired during recovery following discard. Similarly, aerobic metabolism can be important during predator–prey interactions (Killen *et al.,* 2015) and agonistic interactions during territorial disputes (Killen *et al.,* 2014), and so discarded three-striped dwarf cichlids may be more prone to predation or being outcompeted by conspecifics following release, especially if they have experienced air exposure during handling. This decrease in the competition ability could be especially important for males that compete for territories in *Apistogramma* spp. (Rodrigues *et al.,* 2012). Interestingly, there were also several instances where individuals showed a dramatic increase in estimated aerobic scope following an experimental treatment, such that the values for AS_remain_ exceeded 100%. In these cases, it is likely that individuals experienced a short-term bradycardia following the acute stressor (Brijs *et al.,* 2019), causing oxygen uptake to be very low during this brief initial period. Overall, additional research is needed to understand the exact ecological consequences of the reduction in available aerobic scope that occurs during the recovery from discard in this species. Also notable is that several ornamental fish species are capable of air-breathing (e.g. *Corydoras* spp.; Pineda *et al.,* 2020). While the ability to breathe air could reduce the effects of air exposure on these species, any excess post-discard oxygen demand could also cause individuals to increase their air exposure, potentially exposing them to additional predation risk from aerial or terrestrial predators (Kramer, 1987; Pineda *et al.,* 2020).

The metabolic rates of individual three-striped dwarf cichlids decreased quickly from the disturbances associated with capture and discard events, despite there being a large initial increase in aerobic metabolism that could temporarily constrain aerobic scope. In most treatments, the integrated increase in energy expenditure was 10–18% above maintenance over the first 6–15 hours post-treatment (Table 2). These values are likely to be within or close to the routine energy expenditure that these fish display while performing their regular behaviours and digestion, and suggests that the physiological response to handling during capture and release does not elicit a strong degree of additional energy expenditure beyond what fish would normally experience. It is noteworthy, however, that the O_2_ uptake of fish exposed to netting +60 seconds or air exposure remained elevated compared to the other treatments for ~6 hours post-treatment, which resulted in a relatively higher integrated energy expenditure (24% above maintenance) and a constrained aerobic scope during this time. Interestingly, between 30 and 120 minutes of recovery, the MO_2_ of females in the control treatment tended to be higher than that of fish exposed to the intermediate handling treatments (netting and netting +30 seconds air exposure). It is possible that fish receiving the handling stressors were exhibiting reduced spontaneous activity during this time, which would have contributed to a reduction in oxygen uptake relative to the control treatment. Similarly, males exposed to netting +60 seconds of air exposure showed a gradual reduction in MO_2_ and increase in AS_remain_, which eventually surpassed the other treatments by ~11 hours post-treatment, possibly also due to a suppression of activity following recovery. While general activity is limited within the respirometry chambers, fish are still able to turn, swim the length of the chamber (~1–2 body lengths), move their pectoral and caudal fins and perform other small movements. Following bouts of anaerobic metabolism, such as that occurring during burst-type exercise or air exposure in fish, there can be depletions in muscular glycogen, ATP and phosphagens, changes in blood and intracellular muscle pH and various disturbances to ion balance in blood and tissues (Kieffer, 2000; Killen *et al.,* 2003; Holder *et al.,* 2022). Alone or in combination, these factors may reduce the motivation or ability to engage in spontaneous activity during recovery, even if whole-animal oxygen uptake appears to have recovered. It is also possible that activity was reduced to facilitate an increase in AS_remain_ for other aerobic physiological processes, but the exact nature of the interactions among AS_remain_, cellular-level recovery and behaviour require more detailed study. In addition, while most recovery of oxygen uptake of three-striped dwarf cichlids occurs rapidly from the handling stressors encountered during a typical capture-and-discard sequence, further work is needed to determine if there are additional forms of physiological or behavioural disturbance that persist further throughout the recovery period.

Larger individuals showed the greatest increase in MO_2_ immediately post-treatment and recovered MO_2_ more slowly post-treatment. It is not surprising that fish with a greater body mass had a higher IMR, because they require more oxygen to support their increased biomass. However, larger fish also showed a greater increase in IMR in the treatments with air exposure, relative to their own IMR in the control treatment, indicating that larger fish were more sensitive to handling, especially in treatments involving air exposure. Prior work, with other species in the context of recreational fisheries, has found that larger individuals can show greater disturbances to osmoregulatory status, muscle and plasma lactate and oxygen uptake following burst-type activity and air exposure, and take longer to recover than smaller individuals (Clark *et al.,* 2012; Gingerich and Suski, 2012). The reasons for this are not known, but larger individuals may produce more whole-body lactate due to a higher total mass of predominantly anaerobic white muscle, which could then take longer to metabolize in larger fish, perhaps due to the allometric scaling of metabolic rate with body size and potentially reduced activities of key metabolic enzymes (Childress and Somera, 1990; Davies and Moyes, 2007). Larger fish also experience a greater osmoregulatory disturbance following exercise and/or air exposure (Clark *et al.,* 2012; Gingerich and Suski, 2012), which can be energetically costly to recover (Bœuf and Payan, 2001). Regardless of the exact cause, disproportionate effects of handling stress on larger individuals could alter the balance of size-based dominance hierarchies if the capacity for aggression is limited during recovery, or larger and more fecund females could experience adverse effects on reproduction as compared to smaller females. Overall, the causes and consequences of size-based differences in sensitivity to the stressors encountered during capture and discard is an area that requires further investigation.

Males showed a delayed recovery of MO_2_ and AS_remain_ post-handling as compared to females when exposed to netting +60 seconds of air exposure, with the average AS_remain_ of males being <75% until at least 4 hours post-stress. This contrasts with work with sockeye salmon showing that females have a slower recovery following capture-related stressors (Eliason *et al.,* 2020), perhaps because sockeye salmon were examined while during a spawning migration while egg-bearing, while the females in our study did not appear to be carrying eggs and were not in spawning condition. The reason for this sex-based difference in the present study is unclear, but an increase in anaerobic metabolism during handling in males could lead to an elevated oxygen uptake during recovery, as aerobic metabolism is used to fuel the processing of accumulated lactate (Richards *et al.,* 2002). It is possible that males have a higher capacity for anaerobic metabolism, especially if bursts of anaerobic swimming are used more frequently by males during agonistic interactions. It is notable that *Apistogramma* spp. are generally found in hypoxia waters, and so the effects of hypoxia on recovery from handling would be important to measure directly in this taxa. Males have been observed to have a higher capacity for anaerobic metabolism in other fish species (Schoenebeck and Brown, 2012), and further research examining the activity of lactate dehydrogenase or changes in whole-body lactate post-stress in dwarf cichlids would provide more insight into this area. Hormonal signalling also plays a complex role in eliciting the autonomic stress response, possibly contributing to differences in stress responsiveness between males and females (Campbell *et al.*, 2021). Although the exact causes require further study, any asymmetry in the sensitivity of females and males to handling stressors could cause differential effects on reproduction. For example, three-striped dwarf cichlids display biparental care, with the female tending to guard the immediate location around the nest while the male patrols the periphery of the territory (Mendes *et al.,* 2021). Wild *Apistogramma* spp. breed year-round, and so fishing and breeding will overlap. Assuming brooding fish can find their nest territory after being discarded, compromised defences may result in nest predation or abandonment, like observations in recreational fisheries (Suski *et al.,* 2003). Moreover, although effects of the most severe stress treatment on females were reduced as compared to males, female three-striped dwarf cichlids are known to engage in mouthbrooding behaviour (Mendes *et al.,* 2021), and so any increases in oxygen demand could reduce the ability to engage in this important form of parental care given that mouthbrooding is both energetically costly and may constrain ventilation (Reardon and Chapman, 2010).

In summary, our results suggest that following discard after capture, three-striped dwarf cichlids experience an immediate limitation in the aerobic scope available for oxygen-consuming physiological processes. While this constraint is likely to only last until ~30 minutes post-release, during this time individuals would be expected to show an impaired ability to escape predators, find and process food and defend territories, though the exact ecological effects of this transient decrease in aerobic capacity require further study. In addition, larger individuals and males experienced a slower rate of recovery after handling, with modest effects on males being detectable for several hours following the most severe stressor treatment. Additional work could also examine the physiological response of fish captured in actual ornamental fisheries, to provide a more complete picture of the sublethal effects that may occur. It is possible, for example, that fish in an actual fishery could be exposed to air for a longer period during sorting or even be captured multiple times in rapid succession when a netting is repeatedly performed in an area. Still, the results here may be transferable to other *Apistogramma* species that are frequently targeted in the ornamental fish trade and serve as a basis for additional comparative research.

While a physiological approach has been useful for informing regulations and practitioner ethics for other fisheries sectors, and especially for catch-and-release practises in recreational fisheries (Cooke *et al.,* 2013), much more work is required to devise and implement best-practise guidelines for fish collection in the Amazonian ornamental fishery based on physiological data. The current study is the first to explore the proximate effects of discard in this important yet overlooked fishery. Much more data is needed to gain a complete understanding of the factors at play, including the modulating effects of environmental variables on recovery from handling (e.g. temperature and hypoxia, which can be extreme in the habitats where *Apistogramma* spp. are found), species-specific differences and ecological consequences. We encourage further work in this area, coupled with increased interaction with ornamental fishers, to facilitate a path towards increasingly sustainable ornamental fisheries that have minimal ecological impacts.

## Supplementary Material

Web_Material_coad105

## References

[ref1] Arlinghaus R , Cooke SJ, Lyman J, Policansky D, Schwab A, Suski C, Sutton SG, Thorstad EB (2007) Understanding the complexity of catch-and-release in recreational fishing: an integrative synthesis of global knowledge from historical, ethical, social, and biological perspectives. Rev Fish Sci 15: 75–167. 10.1080/10641260601149432.

[ref2] Bates D , Mächler M, Bolker B, Walker S (2015) Fitting linear mixed-effects models using lme4. J Stat Softw 67: 2015. 10.18637/jss.v067.i01.

[ref3] Bœuf G , Payan P (2001) How should salinity influence fish growth? Comparative Biochemistry and Physiology Part C: Toxicology & Pharmacology 130: 411–423. 10.1016/S1532-0456(01)00268-X. 11738629

[ref4] Borges AKM , Oliveira TPR, Rosa IL, Braga-Pereira F, Ramos HAC, Rocha LA, Alves RRN (2021) Caught in the (inter)net: online trade of ornamental fish in Brazil. Biol Conserv 263: 109344. 10.1016/j.biocon.2021.109344.

[ref5] Brijs J , Sandblom E, Rosengren M, Sundell K, Berg C, Axelsson M, Gräns A (2019) Prospects and pitfalls of using heart rate bio-loggers to assess the welfare of rainbow trout (Oncorhynchus mykiss) in aquaculture. Aquaculture 509: 188–197. 10.1016/j.aquaculture.2019.05.007.

[ref60] Campbell JH , Dixon B, Whitehouse LM (2021) The intersection of stress, sex and immunity in fishes. Immunogenetics 73: 111–129. 33426582 10.1007/s00251-020-01194-2

[ref6] Campbell MD , Patino R, Tolan J, Strauss R, Diamond SL (2010) Sublethal effects of catch-and-release fishing: measuring capture stress, fish impairment, and predation risk using a condition index. ICES Journal of Marine Science 67: 513–521. 10.1093/icesjms/fsp255.

[ref7] Chabot D , Steffensen JF, Farrell AP (2016) The determination of standard metabolic rate in fishes. J Fish Biol 88: 81–121. 10.1111/jfb.12845. 26768973

[ref8] Childress JJ , Somera GN (1990) Metabolic scaling: a new perspective based on scaling of glycolytic enzyme activities1. Am Zool 30: 161–173. 10.1093/icb/30.1.161.

[ref9] Clark TD , Donaldson MR, Pieperhoff S, Drenner SM, Lotto A, Cooke SJ, Hinch SG, Patterson DA, Farrell AP (2012) Physiological benefits of being small in a changing world: responses of coho salmon (Oncorhynchus kisutch) to an acute thermal challenge and a simulated capture event. PloS One 7: e39079. 10.1371/journal.pone.0039079. 22720035 PMC3374769

[ref10] Clark TD , Sandblom E, Jutfelt F (2013) Aerobic scope measurements of fishes in an era of climate change: respirometry, relevance and recommendations. J Exp Biol 216: 2771–2782. 10.1242/jeb.084251. 23842625

[ref11] Cohen FPA , Valenti WC, Calado R (2013) Traceability issues in the trade of marine ornamental species. Rev Fish Sci 21: 98–111. 10.1080/10641262.2012.760522.

[ref12] Cook KV , Lennox RJ, Hinch SG, Cooke SJ (2015) FISH out of WATER: how much air is too much? Fisheries 40: 452–461. 10.1080/03632415.2015.1074570.

[ref13] Cooke SJ , Donaldson MR, O’connor CM, Raby GD, Arlinghaus R, Danylchuk AJ, Hanson KC, Hinch SG, Clark TD, Patterson DA et al. (2013) The physiological consequences of catch-and-release angling: perspectives on experimental design, interpretation, extrapolation and relevance to stakeholders. Fisheries Management and Ecology 20: 268–287. 10.1111/j.1365-2400.2012.00867.x.

[ref14] Cooke SJ , Messmer V, Tobin AJ, Pratchett MS, Clark TD (2014) Refuge-seeking impairments mirror metabolic recovery following fisheries-related stressors in the Spanish flag snapper (Lutjanus carponotatus) on the Great Barrier Reef. Physiol Biochem Zool 87: 136–147. 10.1086/671166. 24457928

[ref15] Cooke SJ , Schramm HL (2007) Catch-and-release science and its application to conservation and management of recreational fisheries. Fisheries Management and Ecology 14: 73–79. 10.1111/j.1365-2400.2007.00527.x.

[ref16] Davies R , Moyes CD (2007) Allometric scaling in centrarchid fish: origins of intra- and inter-specific variation in oxidative and glycolytic enzyme levels in muscle. J Exp Biol 210: 3798–3804. 10.1242/jeb.003897. 17951421

[ref17] Eliason EJ , Dick M, Patterson DA, Robinson KA, Lotto J, Hinch SG, Cooke SJ (2020) Sex-specific differences in physiological recovery and short-term behaviour following fisheries capture in adult sockeye salmon (Oncorhynchus nerka). Can J Fish Aquat Sci 77: 1749–1757. 10.1139/cjfas-2019-0258.

[ref18] Evers H-G , Pinnegar JK, Taylor MI (2019) Where are they all from? – sources and sustainability in the ornamental freshwater fish trade. J Fish Biol 94: 909–916. 10.1111/jfb.13930. 30746721

[ref19] Falco F , Bottari T, Ragonese S, Killen SS (2022) Towards the integration of ecophysiology with fisheries stock assessment for conservation policy and evaluating the status of the Mediterranean Sea. Conservation Physiology 10: coac008. 10.1093/conphys/coac008. 35783348 PMC9245081

[ref20] Food and Agriculture Organisation (FAO) (2016) The state of world fisheries and aquaculture 2016. Contributing to food security and nutrition for all.Food and Agriculture Organisation of the United Nations: Rome, Italy. Retrieved from http://www.fao.org/3/a-i5555e.pdf.

[ref21] Fornberg B (2021) Improving the accuracy of the trapezoidal rule. SIAM Rev 63: 167–180. 10.1137/18M1229353.

[ref22] Fu S-J , Dong Y-W, Killen SS (2022) Aerobic scope in fishes with different lifestyles and across habitats: trade-offs among hypoxia tolerance, swimming performance and digestion. Comp Biochem Physiol A Mol Integr Physiol 272: 111277. 10.1016/j.cbpa.2022.111277. 35870773

[ref23] Gingerich AJ , Suski CD (2012) The effect of body size on post-exercise physiology in largemouth bass. Fish Physiol Biochem 38: 329–340. 10.1007/s10695-011-9510-3. 21614550

[ref24] Halsey LG , Killen SS, Clark TD, Norin T (2018) Exploring key issues of aerobic scope interpretation in ectotherms: absolute versus factorial. Reviews in Fish Biology and Fisheries 28: 405–415. 10.1007/s11160-018-9516-3.

[ref25] Holder PE , Wood CM, Lawrence MJ, Clark TD, Suski CD, Weber J-M, Danylchuk AJ, Cooke SJ (2022) Are we any closer to understanding why fish can die after severe exercise? Fish Fish 23: 1400–1417. 10.1111/faf.12696.

[ref26] Kieffer JD (2000) Limits to exhaustive exercise in fish. Comp Biochem Physiol A Mol Integr Physiol 126: 161–179. 10.1016/S1095-6433(00)00202-6. 10938136

[ref27] Killen SS , Christensen EAF, Cortese D, Závorka L, Norin T, Cotgrove L, Crespel A, Munson A, Nati JJH, Papatheodoulou M et al. (2021) Guidelines for reporting methods to estimate metabolic rates by aquatic intermittent-flow respirometry. J Exp Biol 224: jeb242522. 10.1242/jeb.242522. 34520540 PMC8467026

[ref28] Killen SS , Mitchell MD, Rummer JL, Chivers DP, Ferrari MCO, Meekan MG, McCormick MI (2014) Aerobic scope predicts dominance during early life in a tropical damselfish. Functional Ecology 28: 1367–1376. 10.1111/1365-2435.12296.

[ref29] Killen SS , Reid D, Marras S, Domenici P (2015) The interplay between aerobic metabolism and antipredator performance: vigilance is related to recovery rate after exercise. Front Physiol 6: 111. 10.3389/fphys.2015.00111. 25914648 PMC4391267

[ref30] Killen SS , Suski CD, Morrissey MB, Dyment P, Furimsky M, Tufts BL (2003) Physiological responses of walleyes to live-release angling tournaments. North American Journal of Fisheries Management 23: 1238–1246. 10.1577/M02-164.

[ref31] King TA (2019) Wild caught ornamental fish: a perspective from the UK ornamental aquatic industry on the sustainability of aquatic organisms and livelihoods. J Fish Biol 94: 925–936. 10.1111/jfb.13900. 30671948

[ref32] Köhler A , Hildenbrand P, Schleucher E, Riesch R, Arias-Rodriguez L, Streit B, Plath M (2011) Effects of male sexual harassment on female time budgets, feeding behavior, and metabolic rates in a tropical livebearing fish (Poecilia mexicana). Behav Ecol Sociobiol 65: 1513–1523. 10.1007/s00265-011-1161-y.

[ref33] Kramer DL (1987) Dissolved oxygen and fish behavior. Environ Biol Fishes 18: 81–92. 10.1007/BF00002597.

[ref34] Kuznetsova A , Brockhoff PB, Christensen RHB (2017) lmerTest package: tests in linear mixed effects models. J Stat Soft 82: 1–26. 10.18637/jss.v082.i13.

[ref35] Lee CG , Farrell AP, Lotto A, Hinch SG, Healey MC (2003) Excess post-exercise oxygen consumption in adult sockeye (Oncorhynchus nerka) and coho (O. Kisutch) salmon following critical speed swimming. J Exp Biol 206: 3253–3260. 10.1242/jeb.00548. 12909706

[ref36] McLean S , Persson A, Norin T, Killen SS (2018) Metabolic costs of feeding predictively alter the spatial distribution of individuals in fish schools. Curr Biol 28: 1144–1149.e4. 10.1016/j.cub.2018.02.043. 29576472

[ref37] Meka JM , Margraf FJ (2007) Using a bioenergetic model to assess growth reduction from catch-and-release fishing and hooking injury in rainbow trout, Oncorhynchus mykiss. Fisheries Management and Ecology 14: 131–139. 10.1111/j.1365-2400.2007.00533.x.

[ref38] Mendes G , Ricioli LS, Guillermo-Ferreira R (2021) Behavioral repertoire of biparental care in Apistogramma trifasciata (Pisces: Cichlidae). J Appl Ichthyol 37: 957–962. 10.1111/jai.14226.

[ref39] Militz TA , Kinch J, Foale S, Southgate PC (2016) Fish rejections in the marine aquarium trade: an initial case study raises concern for village-based fisheries. PloS One 11: e0151624. 10.1371/journal.pone.0151624. 26963259 PMC4786313

[ref40] Morozov S , McCairns RJS, Merilä J (2019) FishResp: R package and GUI application for analysis of aquatic respirometry data. Conservation Physiology 7: coz003. 10.1093/conphys/coz003. PMC6364290 30746152

[ref41] Norin T , Clark TD (2016) Measurement and relevance of maximum metabolic rate in fishes. J Fish Biol 88: 122–151. 10.1111/jfb.12796. 26586591

[ref42] Papatheodoulou M , Závorka L, Koeck B, Metcalfe NB, Killen SS (2022) Simulated pre-spawning catch and release of wild Atlantic salmon (Salmo salar) results in faster fungal spread and opposing effects on female and male proxies of fecundity. Can J Fish Aquat Sci 79: 267–276. 10.1139/cjfas-2021-0089.

[ref43] Pineda M , Aragao I, McKenzie DJ, Killen SS (2020) Social dynamics obscure the effect of temperature on air breathing in Corydoras catfish. J Exp Biol 223: jeb222133. 10.1242/jeb.222133. PMC7673363 33097572

[ref44] Prang G (2001) A Caboclo Society in the Middle Rio Negro Basin: Ecology, Economy, and History of an Ornamental Fishery in the State of Amazonas. Wayne State University, Brazil.

[ref45] Prinzing TS , Zhang Y, Wegner NC, Dulvy NK (2021) Analytical methods matter too: establishing a framework for estimating maximum metabolic rate for fishes. Ecol Evol 11: 9987–10003. 10.1002/ece3.7732. 34367554 PMC8328417

[ref46] Raby GD , Clark TD, Farrell AP, Patterson DA, Bett NN, Wilson SM, Willmore WG, Suski CD, Hinch SG, Cooke SJ (2015) Facing the river gauntlet: understanding the effects of fisheries capture and water temperature on the physiology of coho salmon. PloS One 10: e0124023. 10.1371/journal.pone.0124023. 25901952 PMC4406555

[ref47] Raby GD , Packer JR, Danylchuk AJ, Cooke SJ (2014) The understudied and underappreciated role of predation in the mortality of fish released from fishing gears. Fish Fish 15: 489–505. 10.1111/faf.12033.

[ref48] Reardon EE , Chapman LJ (2010) Hypoxia and energetics of mouth brooding: is parental care a costly affair? Comp Biochem Physiol A Mol Integr Physiol 156: 400–406. 10.1016/j.cbpa.2010.03.007. 20227513

[ref49] Richards JG , Heigenhauser GJF, Wood CM (2002) Lipid oxidation fuels recovery from exhaustive exercise in white muscle of rainbow trout. American Journal of Physiology-Regulatory, Integrative and Comparative Physiology 282: R89–R99. 10.1152/ajpregu.00238.2001. 11742827

[ref50] Rodrigues RR , Zuanon J, Del-Claro K, Carvalho LN (2012) Reproductive behavior of the Amazonian dwarf cichlid Apistogramma hippolytae Kullander, 1982: offsetting costs and benefits. acta ethologica 15: 47–53. 10.1007/s10211-011-0107-8.

[ref51] Römer U (2021) Baensch/Mergus Cichlid Atlas. Mergus, Berlin.

[ref52] Sandblom E , Gräns A, Axelsson M, Seth H (2014) Temperature acclimation rate of aerobic scope and feeding metabolism in fishes: implications in a thermally extreme future. Proceedings of the Royal Society B: Biological Sciences 281: 20141490. 10.1098/rspb.2014.1490. PMC4211447 25232133

[ref53] Schoenebeck CW , Brown ML (2012) Does anaerobic activity differ seasonally or between sexes in yellow perch populations? Trans Am Fish Soc 141: 199–203. 10.1080/00028487.2012.655119.

[ref54] Schreck CB , Tort L (2016) 1 - The concept of stress in fish. In CB Schreck, L Tort, AP Farrell, CJ Brauner, eds, Fish Physiology. London: Elsevier, pp. 1–34.

[ref55] Suski CD , Svec JH, Ludden JB, Phelan FJS, Philipp DP (2003) The effect of catch-and-release angling on the parental care behavior of male smallmouth bass. Trans Am Fish Soc 132: 210–218. 10.1577/1548-8659(2003)132<0210:TEOCAR>2.0.CO;2.

[ref56] Svendsen MBS , Bushnell PG, Steffensen JF (2016) Design and setup of intermittent-flow respirometry system for aquatic organisms. J Fish Biol 88: 26–50. 10.1111/jfb.12797. 26603018

[ref57] Tribuzy-Neto IA , Beltrao H, Benzaken ZS, Yamamoto KC (2020) Analysis of the Ornamental Fish Exports from the Amazon State. Boletim do Instituto de Pesca, Brazil, p. 46

[ref58] Zahl IH , Samuelsen O, Kiessling A (2012) Anaesthesia of farmed fish: implications for welfare. Fish Physiol Biochem 38: 201–218. 10.1007/s10695-011-9565-1. 22160749

[ref59] Zuur A , Ieno E, Walker N, Saveliev AA, Smith GM (2009) Mixed effects models and extensions in ecology with R (2009 ed.). Springer, New York.

