# Supplementary material for: Capture and discard practises associated with an ornamental fishery affect the metabolic rate and aerobic capacity of three-striped dwarf cichlids Apistogramma trifasciata
Source: Conserv Physiol. 2024 Jan 27;12(1):coad105. doi: 10.1093/conphys/coad105 (PMC10823353; doi:10.1093/conphys/coad105)
Supplement: Web_Material_coad105 [file web_material_coad105.zip › Ojelade supplemental material.pdf]

Table S1. The details of the respirometry set-up for the estimation of metabolic rates of *Apistogramma trifasciata* using intermittent flow respirometry (extracted from Killen *et al.* 2021).

Information	Details
fish body mass	0.564±0.040g (mean±s.e.m.)
volume of empty respirometer	30.0 (small) or 58.3 (large) mL
chamber mixing	peristaltic pump (Masterflex L/S; Cole-Parmer, Vernon Hills, IL, USA)
ratio of net respirometer volume to animal body mass	approx. 1:78 – 1:130
material of the tube used in mixing circuit	Tygon tubing
volume of tubing in the mixing circuit	14 mL
Declare whether the volume of tubing in the mixing circuit was included in calculations of oxygen uptake	Yes, included
type of oxygen probe and data recording	optodes, FireStingO_2; Pyro Science GmbH, Aachen, Germany
material of the respirometry chambers	glass
sampling frequency of water oxygen concentration	2 seconds
position of the oxygen probe	In the recirculating circuit
flow rate during flushing and recirculation, or confirm that chamber returned to normoxia during flushing	~100 ml/min
flush/closed cycles	2/4 minutes

wait (delay) time excluded from closed measurement cycles	30 secs
describe the frequency and method of probe calibration (for both 0 and 100% calibrations)	0% and 100% at the start of the experiment, then 100% calibration every day throughout the experiment, before running initial blank measurements every day of measurement
mention whether software temperature compensation was used during the recording of water oxygen concentration	Yes, used
temperature during respirometry	28.0 ± 0.1℃ (setpoint ± hysteresis)
how temperature was controlled	Thermostat (TMP-REG; Loligo Systems, Viborg, Denmark) that controls a pump to direct water through a heat exchange coil within a heated reservoir whenever temperature within the bath drops below the setpoint
photoperiod during respirometry	12 h /12 h (dark / light)
describe if the ambient water bath was cleaned and aerated during the measurement of oxygen uptake, and if so, how this was done (e.g. filtration, periodic or continuous water changes)	Water bath was cleaned daily throughout experiments with bleach and water. Water during measurements was aerated and UV-filtered throughout the duration of measurements
Provide volume of the ambient water bath and any associated reservoirs	56 L
Minimum water oxygen level or concentration reached during closed phases	5.5 mg/L

Describe whether chambers were visually shielded from external disturbance	The chambers were visually separated with black corrugated plastic dividers, with the entire water bath then being covered with opaque black plastic.
Number of fish that were measured during a given respirometry trial	11-15
Fish were able to see each other during measurements?	No. The chambers were visually separated with black corrugated plastic dividers, with the entire water bath then being covered with opaque black plastic.
Duration of animal fasting before placement in the respirometer	24 hours
Duration of all trials combined (number of days to measure all animals in the study)	12 days of measures over 24 days
Acclimation time to the laboratory before respirometry measurements	Three weeks
Was background respiration measured?	Yes
The method used to measure background respiration	Firstly, all chambers were measured while empty before and after the measurements with fish (three slopes, with a 4 min closed phase and a 2 min flush phase). Secondly, there was always at least one empty chamber running throughout the entire trial. The magnitude and rate of increase in microbial O2 uptake over time in these empty chambers was used to inform an exponential model of microbial respiration over time in the chambers containing fish, within the bounds set by the before and after measures for each chamber. Model predictions of microbial O2 uptake at each time point throughout each trial, in each chamber, were then subtracted from the total O2 uptake occurring at each

	corresponding time point when the chambers contained fish.
Background respiration: number and duration of slopes measured	Three slopes (4 min closed phase; 2 min flush phase), conducted before and after each trial. Parallel empty chambers (at least one per trial) were also run throughout each trial.
How were changes in background respiration modeled over time?	Exponential (see above for additional detail).
Level of background respiration (e.g. as a percentage of average MO_2)	<20%
Method and frequency of system cleaning	The system was cleaned with bleach (approximately 1 ml per 0.5 L of water) and rinsed with continuous water exchange over 30 minutes before every trial.
Time to reach the beginning of metabolic rate measurements after the introduction of fish to the chamber	Logging of water O2 concentration was immediate after closing of chamber. The first two hours after closure were used to calculate an estimate of RMR over this specific duration. The entire trial was then used to calculate another estimate of RMR over this prolonged duration and SMR (control trials only).
duration over which metabolic rate was estimated	21±2 hours
total number of slopes measured and used to derive metabolic rate	165±3.89 (SD) for entire trial
what value was taken as RMR	Mean value of oxygen uptake over either the first two hours of measurement or the entire trials.
When was MMR measured in relation to RMR?	MMR for each fish was measured on a separate day from the RMR measurements, after all trials for RMR had been conducted.
The method used to measure MMR	MMR was estimated by chasing the fish to exhaustion, then measuring O2 uptake immediately after this point.

Was air exposure added after exercise?	Not for the measurement of MMR, but for the measures of RMR in response to stressors, some treatments included air-exposure.

Table S2. Results of linear mixed models, with separate models run using initial metabolic rate after each experimental treatment (IMR), the remaining available aerobic scope after each treatment (AS_{remain}), and excess post-treatment oxygen uptake. Reference levels for categorical variables are female (sex) and control (treatment).

	estimate	s.e.	d.f.	t	p	R^2_m	R^2_c
**IMR**							
intercept	0.395	0.066	79.39	5.979	<0.0001	0.456	0.587
mass	0.108	0.099	95.29	1.092	0.2777		
sex							
male	-0.043	0.032	31.00	-1.348	0.187		
treatment							
net	-0.044	0.070	96.00	-0.478	0.633		
net+30	-0.034	0.070	96.00	-2.071	0.041		
net+60	-0.058	0.070	96.00	-0.824	0.412		
mass x treatment							
mass:net	0.128	0.115	96.00	1.107	0.271		
mass:net+30	0.424	0.115	96.00	3.679	0.0003		
mass:net+60	0.377	0.115	96.00	3.267	0.001		
**AS_{remain}**							
intercept	59.988	12.274	123.44	4.887	<0.0001	0.181	0.253
log10(mass)	36.725	39.374	117.78	0.933	0.353		
sex							
male	2.165	7.632	31.00	0.284	0.779		
treatment							
net	-5.780	16.564	96.00	-0.349	0.728		
net+30	-55.655	16.564	96.00	-3.360	0.001		
net+60	-65.666	16.564	96.00	-3.964	<0.0001		
log10(mass) x treatment							
log10(mass):net	-10.089	51.267	96.00	-0.197	0.844		
log10(mass) net+30	-140.504	51.267	96.00	-2.741	0.007		
log10(mass):net+60	-132.606	51.267	96.00	-2.587	0.011		
**log10 excess post-treatment oxygen uptake (15 hours)**							
intercept	-0.487	0.083	130.00	-5.89	<0.0001	0.009	0.009
log10(mass)	0.086	0.216	130.00	0.396	0.693		
sex							
male	-0.042	0.069	130.00	-0.606	0.546		
treatment							
net	-0.036	0.088	130.00	0.409	0.683		

net+30	-0.004	0.088	130.00	0.050	0.960		
net+60	-0.037	0.088	130.00	0.418	0.676		
**log10 (excess post-treatment oxygen uptake + 0.1) (6 hours)**							
intercept	-0.645	0.102	130.00	-6.306	<0.0001	0.003	0.003
log10(mass)	0.099	0.267	130.00	0.372	0.710		
sex							
male	0.013	0.086	130.00	-0.152	0.879		
treatment							
net	0.008	0.109	130.00	0.072	0.943		
net+30	0.054	0.109	130.00	0.501	0.617		
net+60	0.189	0.109	130.00	1.746	0.083		

s.e. = standard error; d.f. = degrees of freedom

Table S3. Results of linear mixed models for data collected during the 15 hours following experimental treatments, with separate models run Using oxygen uptake (MO_2) and the remaining available aerobic scope after each treatment (AS_{remain}), and excess post-treatment oxygen uptake. Reference levels for categorical variables are female (sex) and control (treatment).

	estimate	s.e.	d.f.	t	p	R^2_m	R^2_c
**log10 MO_2**							
intercept	-0.381	0.066	127.2	-5.827	<0.0001	0.100	0.932
log10(mass)	0.447	0.223	127.2	1.982	0.049		
sex							
male	0.049	0.072	127.2	0.677	0.499		
treatment							
net	-0.001	0.093	127.2	-0.124	0.902		
net+30	0.119	0.093	127.2	1.280	0.203		
net+60	0.010	0.093	127.2	0.105	0.917		
log10(time+0.1)	-0.055	0.029	20380	-18.490	<0.0001		
log10(mass) x treatment							
log10(mass):net	-0.325	0.319	127.2	-1.020	0.310		
log10(mass):net+30	0.506	0.319	127.2	1.586	0.115		
log10(mass):net+60	-0.265	0.319	127.2	-0.832	0.407		
log10(mass) x log10(time+0.1)	-0.083	0.319	20380	-8.206	<0.0001		
treatment x log10(time+0.1)							
net:log10(time+0.1)	0.011	0.004	20380	2.632	0.009		
net+30:log10(time+0.1)	-0.025	0.004	20380	-6.051	<0.0001		
net+60:log10(time+0.1)	-0.016	0.004	20380	-3.916	<0.0001		
sex x log10(time+0.1)	-0.014	0.003	20380	-4.395	<0.0001		
sex x treatment							
male:net	-0.079	0.102	127.2	-0.774	0.440		
male:net+30	0.084	0.102	127.2	0.819	0.415		
male:net+60	0.015	0.102	127.2	0.142	0.887		
sex x treatment x log10(time+0.1)							

male:net:log10(time+0.1)	0.049	0.005	20380	10.530	<0.0001		
male:net+30:log10(time+0.1)	-0.006	0.005	20380	-1.430	0.153		
male:net+60:log10(time+0.1)	-0.017	0.005	20380	-4.090	<0.0001		
log10(mass) x treatment x log10(time+0.1)							
log10(mass):net:log10(time+01)	0.172	0.014	20380	11.940	<0.0001		
log10(mass):net+30:log10(time+01)	-0.060	0.014	20380	-4.124	<0.0001		
log10(mass):net+60:log10(time+01)	0.043	0.014	20380	2.978	0.003		
**log10 (AS_{remain} + 0.1)**							
intercept	1.860	0.018	286.2	103.558	<0.0001	0.100	0.932
log10(mass)	0.070	0.062	286.2	1.135	0.257		
sex							
male	-0.083	0.020	286.2	-4.179	<0.0001		
treatment							
net	-0.046	0.026	290.4	-1.811	0.071		
net+30	-0.010	0.026	298.0	-3.974	<0.0001		
net+60	0.026	0.026	303.7	0.991	0.323		
log10(time+0.1)	0.043	0.004	20380	10.672	<0.0001		
log10(mass) x treatment							
log10(mass):net	0.093	0.088	297.5	1.059	0.290		
log10(mass):net+30	-0.056	0.088	304.1	-6.291	<0.0001		
log10(mass):net+60	0.319	0.088	297.6	3.614	0.0003		
log10(mass) x log10(time+0.1)	-0.018	0.014	20380	-1.286	0.198		
treatment x log10(time+0.1)							
net:log10(time+0.1)	0.020	0.006	20380	3.546	0.0004		
net+30:log10(time+0.1)	0.039	0.006	20380	6.673	<0.0001		
net+60:log10(time+0.1)	-0.002	0.006	20380	-0.464	0.642		
sex x log10(time+0.1)	0.034	0.004	20380	7.642	<0.0001		
sex x treatment							
male:net	0.201	0.028	296.7	7.099	<0.0001		

male:net+30	-0.047	0.028	299.5	-1.647	0.100
male:net+60	-0.050	0.028	296.8	-1.772	0.070
sex x treatment x log10(time+0.1)					
male:net:log10(time+0.1)	-0.075	0.006	20380	-11.660	<0.0001
male:net+30:log10(time+0.1)	0.014	0.006	20380	2.230	0.025
male:net+60:log10(time+0.1)	0.013	0.006	20380	1.976	0.048
log10(mass) x treatment x log10(time+0.1)					
log10(mass):net:log10(time+01)	-0.024	0.020	20380	-1.181	0.237
log10(mass):net+30:log10(time+01)	0.020	0.020	20380	10.035	<0.0001
log10(mass):net+60:log10(time+01)	-0.089	0.020	20380	-4.435	<0.0001

s.e. = standard error; d.f. = degrees of freedom
